# The Assessment of SF-36 Survey for Quality-of-Life Measurement after Radical Cystectomy for Muscle-Invasive Bladder Cancer: A Systematic Review

**DOI:** 10.3390/diseases12030056

**Published:** 2024-03-16

**Authors:** Vlad Barbos, Bogdan Feciche, Silviu Latcu, Alexei Croitor, Vlad Dema, Razvan Bardan, Flaviu Ionut Faur, Tudor Mateescu, Dorin Novacescu, Gherle Bogdan, Alin Adrian Cumpanas

**Affiliations:** 1 Doctoral School, Department of General Medicine, “Victor Babes” University of Medicine and Pharmacy Timisoara, 300041 Timisoara, Romania; vlad.barbos@umft.ro (V.B.); silviu.latcu@umft.ro (S.L.); alexei.croitor@umft.ro (A.C.); vlad.dema@umft.ro (V.D.); tudor.mateescu@umft.ro (T.M.); 2 Department of Urology, Emergency County Hospital Oradea, Strada Gheorghe Doja 65, 410169 Oradea, Romania; 3 Department XV, Discipline of Urology, “Victor Babes” University of Medicine and Pharmacy, 300041 Timisoara, Romania; razvan.bardan@umft.ro (R.B.); cumpanas.alin@umft.ro (A.A.C.); 4 Second Surgery Clinic, Timisoara Emergency County Hospital, 300723 Timisoara, Romania; flaviu.faur@umft.ro; 5 Department X, Discipline of General Surgery, “Victor Babes” University of Medicine and Pharmacy, 300041 Timisoara, Romania; 6 Department II, Discipline of Histology, “Victor Babes” University of Medicine and Pharmacy, 300041 Timisoara, Romania; novacescu.dorin@umft.ro; 7 Angiogenesis Research Center, “Victor Babes” University of Medicine and Pharmacy, 300041 Timisoara, Romania; 8 Doctoral School of Biological and Biomedical Sciences, University of Oradea, 410081 Oradea, Romania; gherlebogdandaniel@gmail.com

**Keywords:** quality of life, oncology, urology

## Abstract

This study presents a systematic review of the literature on individuals’ health-related quality of life (HRQoL) following radical cystectomy for muscle-invasive bladder cancer (MIBC), utilizing the Short Form-36 Health Survey (SF-36) as a primary assessment tool. The review was designed as an exhaustive literature search across three major databases including PubMed, Scopus, and Embase up to December 2023, using the PRISMA guidelines. The selection process refined 2281 identified articles down to 11 studies meeting our inclusion criteria. These studies encompassed a diverse demographic and clinical profile of 774 participants, with follow-up durations ranging from 3 to 130 months, thereby offering insights into both short-term and long-term HRQoL outcomes. The results highlighted significant alterations in individuals’ HRQoL across various domains post-radical cystectomy. Notably, the Physical Functioning (PF) and Bodily Pain (BP) domains generally scored higher, indicating a moderate to high perceived physical health status. However, the Role Physical (RP) and Role Emotional (RE) domains showed variability, reflecting the challenges in daily role fulfillment and emotional adjustment post-surgery. A marked variability in physical recovery was observed, with studies reporting significant differences in PF and RP scores between patient groups. The General Health (GH) and Vitality (VT) domains sometimes reflected perceived deteriorations, whereas the Mental Health (MH) scores suggested that many patients maintained or achieved high levels of well-being post-operatively. The conclusions drawn from this systematic review underscore the profound and multi-faceted impact of radical cystectomy on HRQoL, varying widely between studies, being influenced by geographic factors, surgical methods, and the time of evaluation. The findings emphasize the necessity for holistic patient care approaches that address both physical and emotional rehabilitation, aiming to improve HRQoL outcomes.

## 1. Introduction

According to the World Health Organization, bladder cancer accounts for approximately 3% of all cancer cases worldwide, with muscle-invasive bladder cancer (MIBC) representing a significant proportion of these diagnoses [1,2]. The assessment of health-related quality of life (HRQoL) has become a critical endpoint in the management and evaluation of patients undergoing radical cystectomy for MIBC [3,4]. Radical cystectomy, a mainstay treatment for MIBC, involves the surgical removal of the bladder, often necessitating urinary diversion, which can significantly impact a patient’s lifestyle, psychological well-being, and overall quality of life [5,6,7,8]. Given the invasive nature of this procedure and its life-altering implications, evaluating postoperative HRQoL is paramount to providing comprehensive patient care.

Recent studies have highlighted the significance of HRQoL outcomes in patients with bladder cancer [9,10]. For instance, research has shown varying degrees of impact on the quality-of-life domains assessed by various surveying tools, with notable declines in physical functioning and social functioning after medical and surgical treatment [11]. These findings underscore the importance of integrating HRQoL assessment into the postoperative management and follow-up of these patients. Despite the acknowledged utility of multiple quality-of-life assessment tools such as the World Health Organization Quality of Life (WHOQOL) [12], the Karnofsky Performance Scale [13], the Short Form (SF-12 or SF-8) [14], or the Disability-Adjusted Life Year (DALY) [15], each of these tools measure different psychological domains that do not allow a direct comparison between results.

The Short Form-36 (SF-36) survey stands out as one of the most widely utilized instruments for measuring HRQoL across a broad range of diseases, including cancer [16]. This multidimensional questionnaire evaluates eight domains of health, encompassing physical functioning, role limitations due to physical health problems, bodily pain, general health perceptions, vitality (energy/fatigue), social functioning, role limitations due to emotional problems, and mental health (psychological distress and psychological well-being).

The literature on individuals’ HRQoL post-radical cystectomy, particularly using the SF-36 survey, presents a fragmented view, with studies varying in methodology, sample size, and follow-up duration [17]. This variability complicates the task of drawing comprehensive conclusions about the HRQoL trajectory following surgery for MIBC. Therefore, this study aimed to systematically review changes in individuals’ HRQoL using the SF-36 survey in MIBC patients after radical cystectomy. The objective was to evaluate the surgery’s impact on individuals’ HRQoL based on the SF-36 domains (primary endpoint), and identify differences between different surgical procedures such as ileal conduit and orthotopic neobladder as factors for HRQoL deterioration and as secondary endpoints.

## 2. Materials and Methods

### 2.1. Protocol and Registration

To achieve a thorough and exhaustive literature search, we utilized several key electronic databases, including PubMed, Scopus and Embase. The temporal scope of our search was designed to capture studies published up to December 2023, ensuring the inclusion of the most recent research findings.

The search protocol was formulated to cover an extensive range of keywords and phrases directly relevant to our study’s objectives, focusing on the nuanced aspects of bladder cancer management, particularly after radical cystectomy. The keywords and phrases selected included the following: “muscle-invasive bladder cancer (MIBC)”, “radical cystectomy outcomes”, “SF-36”, “Short Form-36 Health Survey”, “quality of life assessments in oncology”, “QoL post-cystectomy”, “health-related quality of life metrics”, “HRQoL after bladder removal”, “postoperative recovery and QoL”, “urinary diversion techniques”, “neobladder versus ileal conduit outcomes”, “psychosocial effects of bladder cancer surgery”, “physical and emotional rehabilitation post-cystectomy”, “social reintegration after bladder cancer”, “long-term survivorship issues”, “patient-reported outcome measures in uro-oncology”, “adjustment to life post-bladder cancer surgery”, “sexual function after radical cystectomy”, “bladder cancer follow-up care”, “oncological surveillance post-radical cystectomy”, “complications associated with urinary diversions”, and “quality of life evolution over time post-surgery”.

The keywords search strings were meticulously combined using Boolean operators to ensure a thorough retrieval of the literature (“Bladder Neoplasms” [MeSH] OR “muscle-invasive bladder cancer” OR “MIBC”) AND (“Cystectomy” [MeSH] OR “radical cystectomy”) AND (“Quality of Life” [MeSH] OR “SF-36” OR “Short Form-36 Health Survey”) AND (“Quality of Life” OR “life quality” OR “QoL” OR “health-related quality of life” OR “HRQoL”) AND (“Urinary Diversion” [MeSH] OR “urinary diversion” OR “neobladder” OR “ileal conduit” OR “continent urinary reservoir”) AND (“Rehabilitation” [MeSH] OR “physical rehabilitation” OR “physical functioning” OR “emotional well-being” OR “social functioning” OR “psychological adaptation” OR “patient-reported outcomes” OR “social support”) AND (“Sexual Dysfunction, Physiological” [MeSH] OR “sexual function” OR “body image”) AND (“Follow-Up Studies” [MeSH] OR “postoperative care” OR “oncological surveillance” OR “survivorship care”) AND (“Patient Satisfaction” [MeSH] OR “patient satisfaction” OR “treatment outcome” OR “surgical outcomes”).

In adherence to the Preferred Reporting Items for Systematic Reviews and Meta-Analyses (PRISMA) guidelines [18], a structured, transparent, and reproducible approach to the methodology was ensured. Additionally, to promote the transparency and accessibility of our research process and findings, the review has been registered with the Open Science Framework (OSF) to ensure open access to our methodology and findings, with the registration code osf.io/evcju.

The PICO framework of the study was as follows: Population (P): Patients with muscle-invasive bladder cancer undergoing radical cystectomy; Intervention (I): Evaluation of HRQoL using the SF-36 survey; Comparator (C): Different types of urinary diversion techniques and rehabilitation strategies as mentioned in the studies (e.g., ileal conduit vs. neobladder, continence vs. incontinence, early vs. late mobilization); Outcome (O): Changes in HRQoL as assessed by the SF-36 survey, measured at various time points post-surgery (ranging from early assessment at 3 months, up to 3–5 years based on survival).

### 2.2. Eligibility Criteria and Definitions

Initial screening involved the removal of duplicate studies, followed by a detailed assessment of abstracts by two independent researchers to ascertain their relevance to the review’s goals and inclusion criteria. Any discrepancies in study selection were resolved through discussion with a third researcher, ensuring a consensus-based approach to study inclusion.

The inclusion criteria were established as follows: (1) Study population: studies must exclusively involve patients who have undergone radical cystectomy for muscle-invasive bladder cancer; (2) Intervention/Exposure: research must focus on the assessment of quality of life using the SF-36 Survey post-radical cystectomy; (3) Outcome measures: studies should provide detailed results on the SF-36 quality-of-life domains; (4) Study design: observational studies, clinical trials, cohort studies, case–control studies, and cross-sectional studies that have a clear and detailed methodology regarding the use of the SF-36 Survey in this patient population should be included; (5) Language and publication status: only peer-reviewed articles published in English that offer explicit insights into the outcomes related to quality-of-life measurement after radical cystectomy for MIBC are to be included.

The exclusion criteria were defined to eliminate studies that did not meet our specific research needs: (1) Non-human studies: any research not involving human participants or focusing on in vitro or animal models was excluded; (2) Lack of specific focus: studies not exclusively examining quality of life using the SF-36 Survey in patients post-radical cystectomy for MIBC were excluded; (3) Incomplete data: research not providing clear, quantifiable outcomes related to the SF-36 quality-of-life domains post-radical cystectomy was excluded; (4) Non-peer-reviewed sources: grey literature, including non-peer-reviewed articles, preprints, conference proceedings, general reviews, commentaries, and editorials, were excluded to ensure the credibility and reliability of the included data.

### 2.3. Definitions

According to the American Urological Association (AUA) and the European Association of Urology (EAU) guidelines, muscle-invasive bladder cancer (MIBC) is defined based on the extent of tumor invasion into or beyond the bladder’s muscle layer [19]. MIBC encompasses stages T2 to T4a, where T2 indicates that the cancer has invaded the muscle layer (muscularis propria), T3 signifies extension through the muscle layer into the perivesical tissue, and T4 involves invasion into adjacent organs such as the prostate, uterus, vagina, or pelvic wall, but not the abdominal wall (T4a), or the cancer’s spread to the abdominal wall (T4b). These guidelines underscore the severity of MIBC and its distinction from non-muscle-invasive bladder cancer (NMIBC), which is confined to the bladder’s inner layers and has not invaded the muscle layer.

Both the AUA and EAU recommend radical cystectomy as the standard of care for patients with localized MIBC (T2-T4a, N0M0) and consider it an option for high-grade T1 disease that is refractory to Bacillus Calmette–Guérin (BCG) therapy. The guidelines advocate for a multidisciplinary approach to treatment, highlighting the importance of accurate staging and the inclusion of neoadjuvant chemotherapy for eligible patients to improve surgical outcomes and overall survival. Radical cystectomy involves the removal of the bladder along with nearby lymph nodes and, depending on the cancer’s spread and the patient’s gender, may also include the removal of surrounding organs. This aggressive treatment strategy reflects the guidelines’ emphasis on managing the advanced stages of bladder cancer with a potentially curative intent, aiming to maximize patient survival while maintaining quality of life.

### 2.4. Data Collection Process

In the context of this systematic review focusing on the assessment of the SF-36 Survey for quality-of-life measurement following radical cystectomy for muscle-invasive bladder cancer, the initial search across databases resulted in a total of 2281 articles. After a screening process, 264 articles were selected for closer examination, while 382 were identified as duplicates and subsequently excluded to streamline the review process. The screening phase involved a detailed evaluation of abstracts to determine their relevance to the study’s objectives and was conducted independently by two reviewers, with any discrepancies resolved through consultation with a third reviewer to maintain the integrity and objectivity of the selection process.

**Figure 1:**
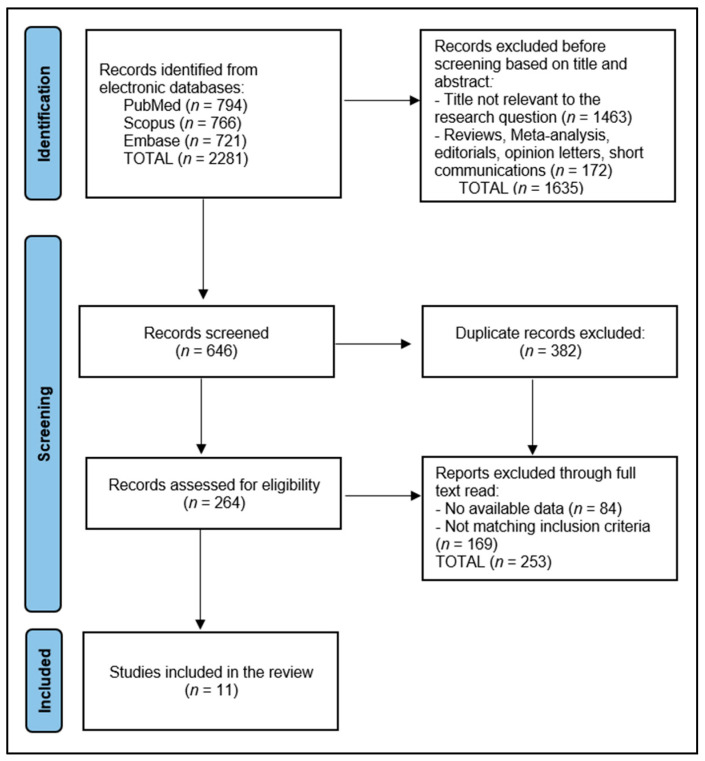
PRISMA Flow Diagram.

Ultimately, 11 articles met the inclusion criteria established for this review, as presented in Figure 1. This selection was based on a comprehensive evaluation of each article’s content, focusing on the utilization of the SF-36 Survey to gauge the quality of life of patients after radical cystectomy in those with muscle-invasive bladder cancer. The included studies were subject to an in-depth data extraction phase, handled by two dedicated researchers in order to gather and synthesize information pertinent to the study design, participant demographics, specifics of the surgical procedures, application of the SF-36 Survey, and the resultant quality-of-life outcomes.

### 2.5. Quality Assessment

To assess the quality of the studies included in our review, we utilized the Newcastle–Ottawa Scale for evaluating cohort studies and the Cochrane Collaboration’s tool for assessing randomized trials [20]. Two researchers independently conducted the evaluations, assigning scores that defined the studies’ quality as either low, medium, or high. This method facilitated a neutral assessment of the literature under review, providing a solid foundation for our systematic analysis.

## 3. Results

### 3.1. Study Characteristics

The systematic review included a total of 11 studies, as outlined in Table 1 [21,22,23,24,25,26,27,28,29,30,31]. These studies were conducted across a variety of countries, including Japan, China, the United Kingdom, Italy, the United States, Ukraine, and Turkey, reflecting a global interest in evaluating the quality of life of patients following radical cystectomy for muscle-invasive bladder cancer. Notably, the research designs primarily consisted of prospective cohort studies [21,22,24,26,27], with a few cross-sectional studies [23,25,28,29,30] and one randomized trial [31].

The quality of evidence presented in these studies varied, with two studies (Autorino et al. [24] and Takenaka et al. [26]) being rated as high, indicating robust research designs and analysis methods. Three studies were assessed as medium quality (Yoneda et al. [27], Miyake et al. [28], Winters et al. [30], and Vermişli et al. [31]), suggesting a moderate level of evidence reliability, while the remaining six studies were categorized as low quality, pointing to potential limitations in their research methodologies or analyses.

### 3.2. Participants’ Characteristics

Table 2 delineates the participant characteristics across the 11 studies in our systematic review, which examined the impact of radical cystectomy on individuals’ quality of life using the SF-36 Survey. The aggregate number of participants across these studies was 774, showcasing a diverse demographic and clinical profile. The participants’ age ranged widely, with a mean age of 58.5 years in the study by Hara et al. [21] to 77 years in the study by Winters et al. [30], indicating the predominant involvement of middle-aged to older adults. The studies predominantly featured male participants, reflective of the higher incidence of muscle-invasive bladder cancer in men, with the exception of Miyake et al. [28], which uniquely included only female participants.

The designs of the participant groups varied, comparing outcomes between those with ileal conduits and neobladders, those experiencing continence versus incontinence post-surgery, and even a comparison between bladder cancer patients and colorectal cancer patients to assess differences in their quality of life. The time of evaluation or follow-up post-surgery ranged significantly from as short as 3 months in the studies by Stakhovskyi et al. [29] and Vermişli et al. [31] to as long as 130 months (median) in the study by Hara et al. [21], indicating both short-term and long-term assessments of quality of life. This variance in follow-up periods allowed for a nuanced understanding of how individuals’ quality of life evolves over time following radical cystectomy. The quality of evidence, as inferred from the study design, suggested a mix of high, medium, and low-quality studies, with two studies (Autorino et al. [24] and Takenaka et al. [26]) rated as high quality, providing a robust foundation for evaluating the impact of radical cystectomy on individuals’ quality of life.

### 3.3. Disease Characteristics

The choice between an ileal conduit (non-orthotopic) and a neobladder (orthotopic) was a central theme, revealing distinct preferences and outcomes across the studies. For instance, Hara et al. [21] reported that 43.5% of their cohort underwent ileal conduit diversion, while 56.5% received a neobladder, indicating a balanced distribution between the two diversion types. In contrast, Takenaka et al. [26] exclusively utilized neobladder reconstruction for all 86 patients in their study, highlighting a shift towards orthotopic diversions in certain clinical settings.

The participant’s continence status, as a measure of urinary control post-surgery, varied significantly, with daytime micturition grades ranging from I to III. Autorino et al. [24] found that 59% of patients achieved Grade I continence following neobladder construction, suggesting a high level of urinary control. These data contrast with the 27.9% incontinence rate reported by Takenaka et al. [26], underscoring the variability in postoperative outcomes and the complex nature of patient recovery.

The cancer staging and grading were reported in several studies, with Yang et al. [22] noting that 93.9% of their 82 patients had Transitional Cell Carcinoma (TCC), predominantly in stages T2 (54.9%) and T3 (39.0%). The statistical interpretation of these findings suggests a correlation between the type of urinary diversion and postoperative continence, which directly influences the patient’s quality of life. The preference for neobladder construction, as evidenced in studies by Takenaka et al. [26] and Autorino et al. [24], may reflect the guidelines used during the time of study, as presented in Table 3.

### 3.4. SF-36 Survey Results

Table 4 describes the different HRQoL domains assessed by the SF-36 questionnaire. The study by Yang et al. [22] used a different format to report the results of the SF-36 survey, where a broad spectrum of responses was observed across the eight SF-36 domains, reflecting the complex and multifaceted nature of recovery and adaptation following surgery for MIBC. In comparison, Hara et al. [21] reported relatively high scores in Physical Functioning (PF) and Bodily Pain (BP), with scores of 75% and 76%, respectively, for the ileal conduit group compared to 74% and 73% for the neobladder group, indicating a moderate to high perceived physical health status post-surgery. However, in the Role Physical (RP) domain, a slight decline was noted (63% vs. 58%), suggesting some challenges in daily role fulfillment due to physical health limitations. Autorino et al. [24] and Fujisawa et al. [25] showed similar trends, with scores generally higher in the PF and BP domains but variable across Role Emotional (RE) and Social Functioning (SF), underscoring the emotional and social adjustment processes experienced post-cystectomy.

Significant differences were particularly highlighted in studies such as those by Philip et al. [23], where Physical Functioning showed a marked difference between groups (77% vs. 61%), pointing to the variability in physical recovery among patients. Similarly, Vermişli et al. [31] recorded a substantial disparity in Role Physical (70% vs. 42%), highlighting the profound impact radical cystectomy can have on patients’ perceived role limitations due to physical health.

**Figure 2:**
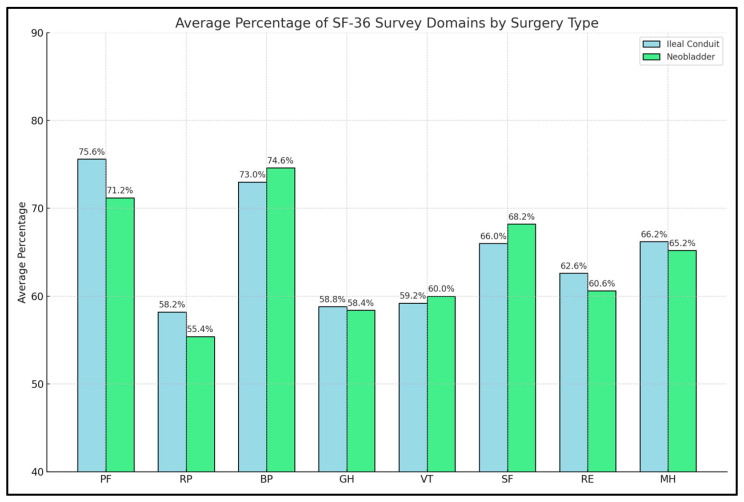
Comparison of aggregate scores of the SF-36 survey domains between patients with ileal conduit and neobladder.

Takenaka et al. [26] and Yoneda et al. [27] provided insights into the lower end of the spectrum in certain domains such as General Health (GH) and Vitality (VT), with scores indicating a perceived deterioration in general health and energy levels. This contrasted with relatively higher scores in Mental Health (MH) in some studies, like that reported by Philip et al. [23] (86% vs. 79%), suggesting that while physical aspects of health may be significantly impacted, many patients maintain or achieve a relatively high level of mental and emotional well-being post-operatively. Overall, even though in some studies patients had a significantly better HRQoL on the SF-36 domains, the pooled data presented in Figure 2 describe an insignificant difference between these two interventions.

## 4. Discussion

### 4.1. Summary of Evidence

The systematic review provides a comprehensive analysis of the impact of radical cystectomy on individuals’ quality of life across multiple domains. The participant characteristics indicate a wide age range and a predominance of male participants, reflective of the demographic most affected by muscle-invasive bladder cancer. The inclusion of studies with various follow-up periods, from 3 to 130 months, offers a broad perspective on the short-term and long-term effects of the surgery on patients’ quality of life. The inclusion of studies spanning nearly two decades in our systematic review acknowledges the dynamic evolution of treatment strategies for muscle-invasive bladder cancer, reflecting the advancements in surgical techniques and postoperative care. This temporal range allows us to capture a broad spectrum of outcomes and adaptations in health-related quality-of-life assessments.

The choice between ileal conduit and neobladder urinary diversions reveals significant differences in patient outcomes and preferences. The shift towards the orthotopic neobladder in certain settings, as seen in the study by Takenaka et al. [26], suggests an evolving landscape in post-cystectomy care. However, the varied continence rates, with some studies reporting high levels of continence and others indicating significant rates of incontinence, point to the complex nature of recovery and the need for further research into optimizing surgical and postoperative strategies to improve these outcomes.

The SF-36 survey results, excluding those presented by Yang et al. [22] for their unique reporting format, reveal noteworthy findings in the Physical Functioning, Bodily Pain, and Mental Health domains. Studies like those by Hara et al. [21] and Fujisawa et al. [25] report relatively high scores in PF and BP, suggesting that patients can retain or regain a good level of physical health post-surgery. However, the variability in the RP and Social Functioning scores across studies highlights the challenges patients may face in daily role fulfillment and social interactions post-cystectomy. These findings emphasize the multifaceted nature of patient recovery, where physical health improvements are seen alongside ongoing challenges in emotional and social domains.

Critically, this review identifies significant disparities in quality-of-life outcomes, particularly in the RE and RP domains, indicating that radical cystectomy has a profound impact on patients’ perceptions of their ability to fulfill daily roles and activities. The studies by Philip et al. [23] and Vermişli et al. [31] particularly highlight these disparities, underscoring the need for targeted interventions to address these aspects of recovery. The variability in scores and the presence of statistically significant differences across studies call for a more nuanced understanding of the factors influencing these outcomes. As the field moves forward, it becomes increasingly clear that addressing the physical aspects of recovery from radical cystectomy is only one part of the equation. Equally important is the need to support patients through the emotional and social challenges posed by such a life-changing procedure, ensuring a holistic approach to care that optimizes the overall quality of life post-surgery.

A recent study by Francolini et al. [32] focused on evaluating the impact of radical cystectomy (RC), continent cutaneous urinary diversion (CCUD), orthotopic neobladder (ONB), and trimodal therapy (TMT) on the quality of life (QoL) of patients with non-metastatic muscle-invasive bladder cancer (MIBC). The findings highlighted a significant advantage of ONB over other methods in terms of Physical Functioning, with a pooled mean standardized difference of −0.73 SD, and Emotional Functioning, with a −0.16 SD difference. TMT showed a trend towards higher mean reported values in the Global Health Score, Physical Functioning, and Role Functioning compared to both RC approaches. While ONB demonstrated a significant benefit in specific QoL subdomains compared to ICUD, no direct comparison was available with TMT. However, the data suggested a potential advantage of TMT over both reconstructive scenarios.

Even though our systematic review analyzed specifically the SF-36 survey results, a multitude of different studies assessed the quality of life among patients after bladder cancer surgery. The study by Catto et al. [10] investigated the health-related quality of life of bladder cancer patients, revealing that almost 70% of participants reported at least one problem in any EQ-5D dimension. Despite variations in the population features, the HRQOL outcomes remained similar across all groups. Notably, the prevalence of problems increased with age and the number of long-term conditions. Sexual dysfunction was a common issue among men, particularly in younger patients and those who underwent radical treatments. Compared to the general population and patients with other pelvic cancers, BC patients exhibited a significantly worse HRQOL, with 69% reporting problems in one or more EQ-5D dimensions versus 51% in the general population.

The same EQ-5D survey was used in a very large cross-sectional study where Mason et al. [33] focused on assessing the QoL among adults surviving bladder cancer 1–5 years post-diagnosis. The survey findings revealed that a significant number of patients across all treatment groups reported experiencing problems in one or more EQ-5D generic domains, with percentages ranging from 59% to 74%. The most frequently reported issue was with usual activities. Furthermore, urinary frequency was common, especially after endoscopy (34–37%) and radiotherapy (44–50%).

In more advanced BC stages, quality of life is viewed as accommodating palliative care, where sleep and rest are the main domains. The study by Tsai et al. [34] utilized the WHOQOL-BREF questionnaire and assessed 109 patients, of which 42 underwent radical cystectomy. The findings revealed that, except for specific items in the physical domain, radical cystectomy did not significantly differ in QoL outcomes compared to bladder-sparing techniques across most domains. Particularly noteworthy was the observation that patients in the advanced stages (III–IV) of cancer reported improved sleep and rest post-cystectomy over a period extending beyond five years, a benefit not observed in stage II patients.

The studies by Ungerer et al. [35] and Clements et al. [8] offer valuable perspectives on the impact of bladder cancer treatments on patients’ QoL, yet with distinct focal points and findings. Ungerer et al. reveal a significant decline in patients’ ability to perform activities of daily living (ADLs) and in their overall health status post-bladder cancer diagnosis, with specific reductions in physical (PCS12) and mental (MCS12) health composite scores (*p* < 0.0001 for both). In contrast, Clements et al. delve into the nuanced recovery patterns following radical cystectomy, showing that physical function scores decrease initially but tend to stabilize or return to baseline within 24 months, while social function improves, particularly among patients with continent diversions. Importantly, Clements et al. also highlight the baseline HRQOL differences between diversion groups, with continent diversion patients reporting better physical, urinary, and sexual functions pre-RC.

Lastly, it is important to acknowledge the existence of many tools for surveying individuals’ quality of life in oncology and, more specifically in the context of this study, the impact of radical cystectomy for MIBC. As there are many surveying tools, there are also multiple management and reconstruction options for MIBC, that, put together, create a large heterogeneity effect. One meta-analysis by Kimura et al. [36] revealed that robot-assisted radical cystectomy resulted in longer operative times but significantly decreased blood loss and a reduction in the need for transfusions compared to open radical cystectomy. Importantly, no significant differences were observed in major postoperative complications within 90 days or in the health-related quality of life outcomes at 3 and 6 months post-surgery between the two techniques. Although non-randomized studies have suggested that robot-assisted radical cystectomy offers benefits in terms of lower complication rates, reduced mortality, and shorter hospital stays, these findings were not corroborated by randomized trials. Similarly, another trial compared open radical cystectomy and robot-assisted radical cystectomy with intracorporeal urinary diversion [37]. At the 1-year mark, both groups reported a significant deterioration in body image and physical and sexual functions. Specifically, open radical cystectomy patients faced more significant impairments in role functioning, with their symptom scales and bowel symptoms worsening. In contrast, those who underwent robot-assisted radical cystectomy reported significant increases in urinary symptoms and problems. These data underscore the differential impact of surgical approaches on HRQoL, with open radical cystectomy associated with broader functional declines and robot-assisted radical cystectomy with specific urinary challenges.

The synthesis of SF-36 survey data in our study highlights a generally favorable health-related quality of life for patients post-radical cystectomy, confirming the SF-36 as a pertinent instrument for evaluating global HRQoL outcomes in this context. Such findings advocate for the SF-36’s efficacy in elucidating the broader health status implications for individuals treated for bladder cancer, underscoring the potential to maintain a considerable level of life quality despite the disease’s significant challenges. In assessing HRQoL in post-cystectomy patients, clinicians and researchers are advised to use the SF-36 survey, as well as complement it with disease-specific instruments for a more comprehensive evaluation.

### 4.2. Limitations

The limitations of this systematic review stem from the inherent heterogeneity and variable quality of the included studies, which complicate the synthesis of findings regarding the impact of radical cystectomy on HRQoL using the SF-36 survey. Despite a rigorous search and selection process, the review is constrained by the small number of studies that met the inclusion criteria, reflecting a limited evidence base. Additionally, variations in the study design, population demographics, and follow-up periods across these studies introduce challenges related to the generalization of results. While the SF-36’s generality allows for broad comparisons across diseases, we recognize its limitation in capturing the disease-specific nuances important to HRQoL in radical cystectomy patients, as those of the European Organization for Research and Treatment of Cancer are more widely preferred. Furthermore, the assessment of study quality highlighted a mix of low to high-quality studies, suggesting that some findings might be influenced by methodological weaknesses, thereby affecting the review’s overall conclusions on HRQoL outcomes post-radical cystectomy for MIBC. Given the limited number of studies included in our systematic review, we chose not to conduct a sensitivity analysis by excluding low-quality studies in order to preserve the size of data and maintain a comprehensive understanding of the field, thereby ensuring that our review reflects the full spectrum of existing research and supports a more inclusive analysis of the topic at hand. Moreover, our review observations indicate variations in HRQoL scores across different regions, hinting at the potential impact of diverse healthcare systems, cultural perceptions of illness and recovery, and access to supportive care services.

## 5. Conclusions

In conclusion, the SF-36 survey is widely used in urological oncology, being among the most comprehensive tools used to assess the plentiful psychological domains. It was observed that radical cystectomy significantly impacts individuals’ HRQoL across multiple domains, with substantial alterations observed in physical functioning, role limitations due to physical health, and general health perceptions. Despite these challenges, many patients exhibit resilience in mental health domains. These insights highlight the critical need for comprehensive care strategies that support both physical and emotional recovery, aiming to enhance the overall HRQoL outcomes for MIBC patients post-radical cystectomy.

## Figures and Tables

**Table 1:** Study characteristics.

Study and Author	Country	Study Year	Study Design	Quality of Evidence
1. Hara et al. [21]	Japan	2002	Prospective cohort	Low
2. Yang et al. [22]	China	2013	Prospective cohort	Low
3. Philip et al. [23]	United Kingdom	2009	Cross-sectional	Low
4. Autorino et al. [24]	Italy	2009	Prospective cohort	High
5. Fujisawa et al. [25]	Japan	2000	Cross-sectional	Low
6. Takenaka et al. [26]	Japan	2010	Prospective cohort	High
7. Yoneda et al. [27]	Japan	2005	Prospective cohort	Medium
8. Miyake et al. [28]	Japan	2012	Cross-sectional	Medium
9. Stakhovskyi et al. [29]	Ukraine	2020	Cross-sectional	Low
10. Winters et al. [30]	United States	2018	Cross-sectional	Medium
11. Vermişli et al. [31]	Turkey	2021	Randomized Trial	Medium

**Table 2:** Characteristics of the participants.

Study Number	Age (Years)	Sex	Number of Patients	Control Group	Time of Evaluation/ Follow-Up
1. Hara et al. [21]	58.5 (mean)	85 (100%) men	85	Ileal conduit vs. Neobladder	Follow-up: 130 months (median)
2. Yang et al. [22]	66.0 (mean)	NR	82	Ileal conduit vs. Neobladder	At 6 months, 1 year and 2 years after surgery
3. Philip et al. [23]	65.5 vs. 73.5	40 (76.9%) men	52	Ileal conduit vs. Neobladder	15 months (median)
4. Autorino et al. [24]	65.9 vs. 63.5	79 (100%) men	79	Ileal conduit vs. Neobladder	>12 months
5. Fujisawa et al. [25]	61.4 vs. 70.6	38 (67.8%) men	56	Ileal conduit vs. Neobladder	31 vs. 44 months
6. Takenaka et al. [26]	62.0 (median)	78 (90.7%) men	86	Continence vs. Incontinence	89 months
7. Yoneda et al. [27]	65.6 (mean)	47 (83.9%) men	56	NR	51.6 months
8. Miyake et al. [28]	63 (median)	32 (100%) women	32	Sigmoid vs. Ileal Neobladder	>12 months
9. Stakhovskyi et al. [29]	58.5 vs. 60.5	NR	40	Ileal conduit vs. Neobladder	3 months
10. Winters et al. [30]	77 (mean)	126 (76%)	166	Bladder vs. Colorectal Cancer	24 months
11. Vermişli et al. [31]	64.8 (mean)	20 (50%)	40	Early vs. Late Mobilization	3 months

NR—Not Reported.

**Table 3:** Disease characteristics.

Study Number	Ileal Conduit (Non-Orthotopic)	Neobladder (Orthotopic)	Status of Continence	Cancer Type/Staging/Grading
1. Hara et al. [21]	37 (43.5%)	Ileum 26 (30.6%) Colon 22 (25.9%)	Daytime micturition: Grade I (52%) Grade II (38%) Grade III (10%)	NR
2. Yang et al. [22]	28 (34.1%)	54 (65.9%)	NR	77 (93.9%) TCC 5 (6.1%) T1 45 (54.9%) T2 32 (39.0%) T3
3. Philip et al. [23]	24 (46.1%)	28 (53.9%)	15% incontinence	NR
4. Autorino et al. [24]	44 (55.7%)	35 (64.3%)	Daytime micturition: Grade I (59%) Grade II (34%) Grade III (7%)	59 (74.6%) T2 13 (25.4%) T3
5. Fujisawa et al. [25]	20 (35.7%)	36 (64.3%)	Daytime micturition: Grade I (53.6%) Grade II (35.7%) Grade III (10.7%)	NR
6. Takenaka et al. [26]	0 (0%)	86 (100%)	27.9% incontinence	NR
7. Yoneda et al. [27]	0 (0%)	56 (100%)	NR	NR
8. Miyake et al. [28]	0 (0%)	Ileum 14 (43.8%) Sigmoid 18 (56.2%)	Spontaneous voiding: Ileum—64.3% Sigmoid—94.4% Daytime micturition: Ileum—91.7% Sigmoid—83.3%	21 (65.6%) T2 9 (28.1%) T3 2 (6.3%) T4
9. Stakhovskyi et al. [29]	20 (50%)	20 (50%)	NR	25 (62.5%) T2 9 (22.5%) T3 6 (15.0%) T4
10. Winters et al. [30]	156 (100%)	0 (0%)	NR	NR
11. Vermişli et al. [31]	40 (100%)	0 (0%)	NR	NR

NR—Not Reported; TCC—Transitional Cell Carcinoma.

**Table 4:** Comparison of HRQoL domains assessed by the SF-36 questionnaire between the analyzed studies.

Study Number **	PF	RP	BP	GH	VT	SF	RE	MH	TOTAL
1. Hara et al. [21]	75% vs. 74%	63% vs. 58%	76% vs. 73%	50% vs. 50% *	55% vs. 55%	52% vs. 46%	62% vs. 66% *	64% vs. 65%	NR
2. Yang et al. [22]	23.0 vs. 24.1	5.7 vs. 5.2	9.2 vs. 10.3	19.6 vs. 11.3 *	16.3 vs. 14.2	7.3 vs. 4.8 *	3.9 vs. 3.1 *	23.1 vs. 15.1 *	112.8 vs. 95.4 *
3. Philip et al. [23]	77% vs. 61% *	68% vs. 59%	78% vs. 79%	73% vs. 68%	61% vs. 62%	79% vs. 79%	84% vs. 79%	86% vs. 79%	NR
4. Autorino et al. [24]	70% vs. 76%	61% vs. 59% *	71% vs. 72%	60% vs. 60%	54% vs. 52%	71% vs. 60% *	63% vs. 52% *	69 vs. 64%	NR
5. Fujisawa et al. [25]	78% vs. 80%	54% vs. 64% *	71% vs. 80%	56% vs. 64%	62% vs. 70%	76% vs. 81%	55% vs. 66% *	71% vs. 76%	NR
6. Takenaka et al. [26]	50% vs. 44%	45% vs. 40%	54% vs. 50%	51% vs. 42% *	53% vs. 50%	50% vs. 41% *	46% vs. 39%	52% vs. 48% *	NR
7. Yoneda et al. [27]	75% vs. 77%	61% vs. 79% *	69% vs. 70%	57% vs. 61%	62% vs. 63%	81% vs. 85%	68% vs. 80% *	70% vs. 75%	NR
8. Miyake et al. [28]	48% vs. 39%	43% vs. 46%	53% vs. 48%	49% vs. 47%	53% vs. 50%	48% vs. 40%	44% vs. 35%	51% vs. 50%	NR
9. Stakhovskyi et al. [29]	78% vs. 65%	45% vs. 37%	69% vs. 69%	55% vs. 50%	64% vs. 61%	52% vs. 75%	49% vs. 40%	41% vs. 42%	NR
10. Winters et al. [30]	53% vs. 51%	42% vs. 38%	50% vs. 56%	52% vs. 51%	51% vs. 48%	70% vs. 66%	66% vs. 62%	73% vs. 70%	NR
11. Vermişli et al. [31]	74% vs. 66%	70% vs. 42% *	49% vs. 53%	64% vs. 38% *	66% vs. 41% *	65% vs. 48% *	73% vs. 45% *	83% vs. 60% *	NR

*—Statistically significant differences; **—Study 2 (Yang et al. [22]) reported data as nominal values instead of percentages; NR—Not Reported; PF—Physical Functioning; RP—Role Physical Functioning; BP—Bodily Pain; GH—General Health; VT—Vitality; SF—Social Functioning; RE—Role Emotional Functioning; MH—Mental Health; Studies 1–5, and 9 report a comparison between ileal conduit vs. neobladder.
